# Causes and Implications of Codon Usage Bias in RNA Viruses

**DOI:** 10.1371/journal.pone.0056642

**Published:** 2013-02-25

**Authors:** Ilya S. Belalov, Alexander N. Lukashev

**Affiliations:** 1 Chumakov Institute of Poliomyelitis and Viral Encephalitides, Russian Academy of Medical Sciences, Moscow, Russia; 2 Institute for Virology, University of Bonn Medical Center, Bonn, Germany; University of Edinburgh, United Kingdom

## Abstract

Choice of synonymous codons depends on nucleotide/dinucleotide composition of the genome (termed mutational pressure) and relative abundance of tRNAs in a cell (translational pressure). Mutational pressure is commonly simplified to genomic GC content; however mononucleotide and dinucleotide frequencies in different genomes or mRNAs may vary significantly, especially in RNA viruses. A series of *in silico* shuffling algorithms were developed to account for these features and analyze the relative impact of mutational pressure components on codon usage bias in RNA viruses. Total GC content was a poor descriptor of viral genome composition and causes of codon usage bias. Genomic nucleotide content was the single most important factor of synonymous codon usage. Moreover, the choice between compatible amino acids (e.g., leucine and isoleucine) was strongly affected by genomic nucleotide composition. Dinucleotide composition at codon positions 2-3 had additional effect on codon usage. Together with mononucleotide composition bias, it could explain almost the entire codon usage bias in RNA viruses. On the other hand, strong dinucleotide content bias at codon position 3-1 found in some viruses had very little effect on codon usage. A hypothetical innate immunity sensor for CpG in RNA could partially explain the codon usage bias, but due to dependence of virus translation upon biased host translation machinery, experimental studies are required to further explore the source of dinucleotide bias in RNA viruses.

## Introduction

Amino acid sequence of proteins is encoded by nucleotide triplets. The majority of organisms use the standard genetic code, with 61 sense codons translated into 20 amino acids. Therefore, most amino acids are encoded by several synonymous codons, which are not used evenly. This codon usage bias (CUB) can have distinct causes and consequences in different organisms. Two major factors are implicated to explain CUB, termed (somewhat ambiguously) translational (selection) and mutational pressure [1]. Translational pressure means preference towards codons that are most suitable for translation in a given context. Evidence for translational pressure towards common codons has been found in certain highly expressed bacterial genes [2]–[4].Use of rare codons was hypothesized to slow down the translation rate to ensure optimal levels of protein expression and proper folding [5], however regulation of translation rate in bacteria might rather rely on specific sequence fragments than simply on rare codons [6]. Moreover, it was shown that a synonymous substitution can result in a protein with different folding and different function, probably by causing ribosome stalling [7]. There are also reports of translational pressure in eukaryotes [8], [9]. While the implications of translational pressure are ubiquitous (reviewed in [10], [11]), a growing body of evidence suggests that it is not the main driver of synonymous codon preference.

Mutational pressure is produced by distinct probability of different substitution types. A predominant factor driving codon usage is believed to be GC content, i.e. the summed relative abundance of G and C nucleotides [12], [13]. Additional factors that might contribute to mutational pressure are deoxycytidine methylation in CpG (C-phosphate-G) dinucleotide context and subsequent deamination that results in C-T substitution [14]. Nucleotide content bias in viral RNA genomes could also be produced by RNA editing by cellular transaminases (e.g. ADAR and APOBEC); however, the extent of such editing of cellular mRNA is still a matter of controversy [15], [16]. Although RNA editing has been speculated to complement to variability of viral genomes [17], no mechanistic proof has been presented, and its role in the evolution of viruses remains to be proven.

Several additional factors of DNA/RNA variation can be classified as selection, but not translational pressure. Firstly, there may be a selection against a sequence pattern that triggers innate immunity, e.g. toll-like receptor 9 (TLR9) by CpG rich bacterial DNA [18], or against a target of immunity effectors. The latter case is exemplified by UpA dinucleotide, targeted by RNAse L [19], which presumably resulted in reduced UpA content in, e.g. hepatitis C virus [20]. Another factor that might constrain synonymous codon usage, especially in RNA viruses, is the secondary RNA structure, either nonspecific RNA folding, presumably to protect it from intracellular defense mechanisms [21], [22], or regulatory structural RNA elements commonly found in viruses within the coding sequence [23], [24]. These forces are hard to distinguish from the mutational pressure because they act above the translation level.

Investigation of CUB source is most commonly performed *in silico* and relies on several approaches, which can be generally classified as host-dependent and host-independent. Host-dependent methods rely upon either total codon usage statistics for an organism and compare it to codon usage in specific genes, or upon tRNA abundance, which can be measured experimentally or extrapolated from the number of different tRNA genes in the genome [25]–[27]. These approaches have limited value in virology because tRNA statistics are available only for a few model organisms. Also, the native host of a virus is often not known, and virus replication commonly takes place only in a specific type of cell, which may have relative tRNA concentrations and codon usage distinct from other tissues [28]. In addition, viral infection can result in inhibition or activation of RNA polymerase III and thus affect transcription of multiple genes, including tRNAs, in an infected cell [29], [30]. The host-independent approach accounts only for uneven frequency of synonymous codons and is commonly expressed as effective number of codons, ENC [31]. This single number theoretically ranges from 61 (all codons used equally) to 20 (only one codon used per amino acid). ENC below 40 is commonly treated as evidence of a strong CUB. It is a robust and organism-independent method that is universally used to evaluate CUB. A statistical correction termed Nc' was subsequently developed to account for genomic GC content [32]. While a notable improvement over the original method, Nc' has its faults and limitations, especially in the case of short analyzed sequences [33]. In addition, Nc' does not explicitly account for mononucleotide and dinucleotide composition bias that may be especially high in viral genomes.

Most RNA viruses display low to moderate CUB, which was attributed mainly to GC and dinucleotide content [34]. Combined GC content was the first measure of genome composition, implemented even before the genetic code was deciphered [35]. While this simple criterion covers the majority of genome composition bias, it does not account for different mononucleotide frequencies. This work investigates the impact of different types of mutation pressure on CUB in RNA viruses and provides an analysis of CUB in common RNA viruses.

## Materials and Methods

Coding sequences of 29 animal RNA viruses representing 12 families (Table 1) were analyzed for codon usage and dinucleotide bias. Reference GenBank records and sequences of prototype strains were preferred. Concatemers of open reading frames (ORFs) were used for viruses with segmented genomes. For viruses with a complex coding strategy concatemers of ORFs, excluding regions with overlapping frames (as described in the corresponding Genbank records), were used. The exact genes used in the study and virus name acronyms are provided in Table 1. Additionally, five representative datasets of common RNA viruses were created. The sequences were chosen from all available complete coding sequences by automatically omitting identical or very similar sequences. The cutoff for dropping similar sequences was selected individually for each virus to produce datasets of a manageable size. The datasets were 69 out of 1525 available complete coding sequences of hepatitis C virus (HCV), all of 39 of hepatitis A virus (HAV) sequences, 74 out of 1283 Dengue 1 sequences, 68 out of 154 human enterovirus C sequences, and 79 out of 12327 segment 2 coding sequences of influenza A virus (PB1 polymerase fragment, not excluding PB1-F2 ORF). Simulated random sequences were created by first generating random values of third-position nucleotide frequencies. The randomization algorithm was designed to generate extreme values to cover all theoretically possible range of values in a reasonably sized dataset. Then 1000 random amino acid sequences were generated using these third-position nucleotide frequencies for synonymous codon choice.

**Table 1:** Virus sequences used in this work.

Virus	Family	Genome polarity	Acronym	Segment	Lenth	GenBank
Lassa virus	Arenaviridae	−	Lassa	N+L	8361	NC_004296, 97
LCMV	Arenaviridae	−	LCMV	N+L	8304	NC_004291, 94
La Crosse virus	Bunyaviridae	−	Bun	S+M+L	7212	NC_004108, 10
Ebola virus	Filoviridae	−	Ebola	AllORFs	13398	AF086833
Influenza A virus	Orthomyxoviridae	−	FluA	AllORFs	3498	NC_002016-23
Influenza B virus	Orthomyxoviridae	−	FluB	AllORFs	3990	NC_002204-11
Influenza C virus	Orthomyxoviridae	−	FluC	AllORFs	3957	NC_006306-12
Thogotovirus	Orthomyxoviridae	−	ThgV	AllORFs	3492	NC_006495, 507
Bovine parainfluenza 3	Paramyxoviridae	−	BPI3	AllORFs	13812	D84095
Measles virus	Paramyxoviridae	−	Measles	AllORFs	13596	K01711
Mumps virus	Paramyxoviridae	−	Mumps	AllORFs	13578	AF201473
Respiratory syncytial virus	Paramyxoviridae	−	RSV	AllORFs	12033	U39661
Rabies virus	Rhabdoviridae	−	Rabies	AllORFs	10800	AB044824
Vesicular stomatitis virus	Rhabdoviridae	−	VSV	AllORFs	10608	J02428
Human astrovirus	Astroviridae	+	HAstV	AllORFs	6633	NC_001943
Norwalk virus	Caliciviridae	+	NoroV	AllORFs	7560	NC_001959
Hepatitis A virus	Picornaviridae	+	HAV	ORF	6684	M14707
Aichi virus	Picornaviridae	+	AiV	ORF	7302	NC_001918
GB virus C	Flaviviridae	+	GBV-C	ORF	8526	AB003292
Yellow fever virus	Flaviviridae	+	YFV	ORF	10233	AF094612
Dengue 1 virus	Flaviviridae	+	Den1	ORF	10176	AF180818
West Nile virus	Flaviviridae	+	WNV	ORF	10299	AF206518
Hepatitis C virus	Flaviviridae	+	HCV	ORF	9030	AJ000009
Hepatitis E virus	Hepeviridae	+	HEV	AllORFs	6738	NC_001434
Western equine encephalomyelitis virus	Togaviridae	+	WEEV	AllORFs	11109	AF214040
Rubella virus	Togaviridae	+	Rubella	AllORFs	9576	AF188704
Rotavirus C	Reoviridae	DS	RotaC	VP2+Pol	5922	NC_007546, 47
Human immunodeficiency virus	Retroviridae	+	HIV	Pol+Gag	4083	NC_001802
Human T-lymphotropic virus 1	Retroviridae	+	HTLV1	Pol+Gag	3630	NC_001436

DS, double stranded.

The effective number of codons, ENC [31], was calculated with the “chips” module in the EMBOSS package (http://emboss.sourceforge.net/). ENC of 20 corresponds to extreme bias (only one synonymous codon per amino acid), ENC of 61 indicates absence of bias. Python 2.6 scripts were developed for dinucleotide content analysis (dint-stat), sequence randomization and shuffling (cub-stat) (available at http://www.poliomielit.ru/images/doc/enc-stat.rar). The difference between these two approaches is that shuffling moves sequence fragments (e.g. dinucleotides) from one place in a sequence to another, while randomization first counts sequence statistics, and then generates a random sequence aiming to reach these values. As generation of sequences is random, they never have precisely the same composition as the original sequence. A total of 1000 scrambled replicates were analyzed for each virus and each algorithm. The algorithms of these scripts are summarized below.

### N_3_ correction

**Figure 1:**
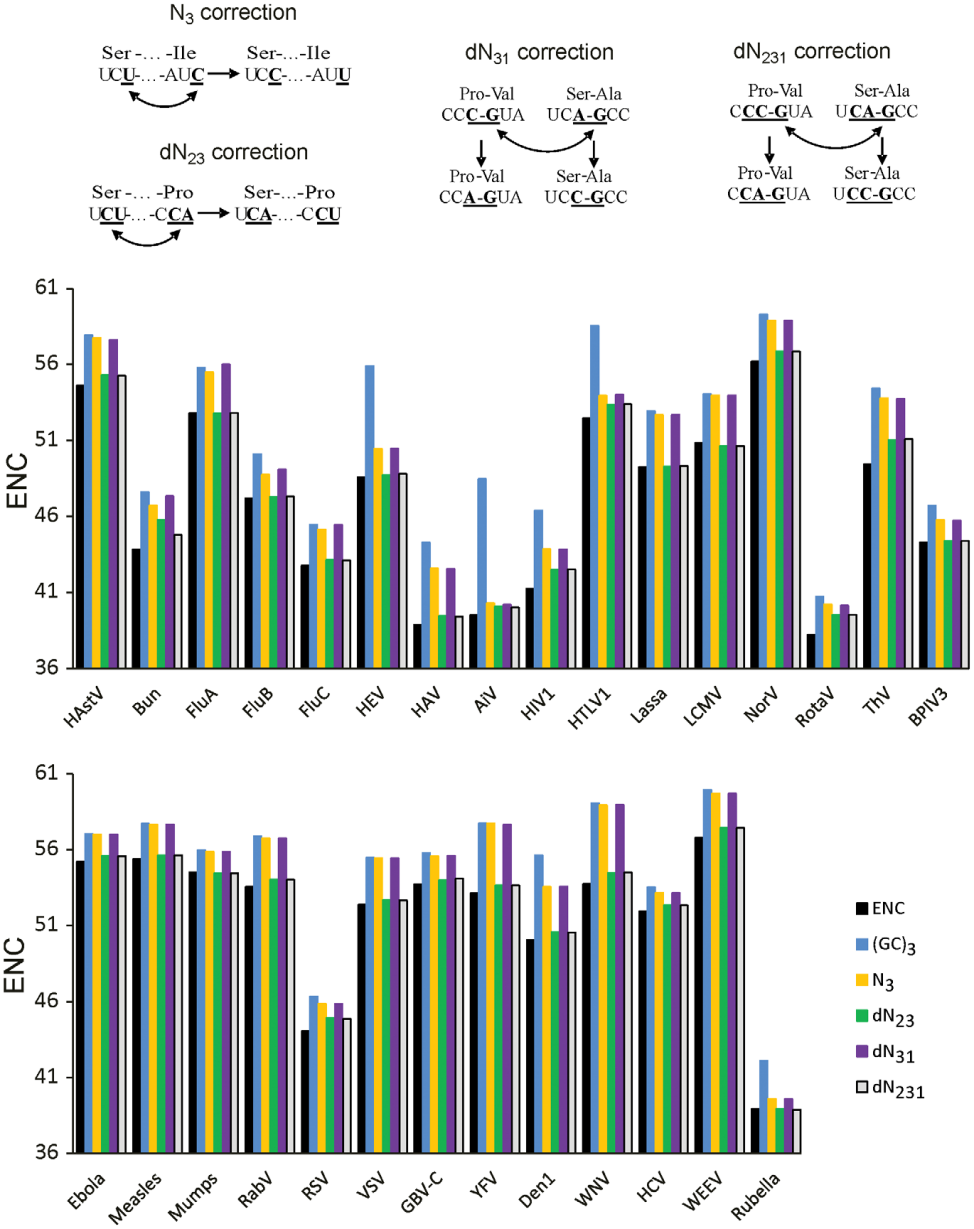
Effective number of codons in the original and shuffled sequences. (A) Explanation of shuffling algorithms. (B) Effective number of codons in the original virus sequence and the mean ENC of 1000 sequences randomized or scrambled using different algorithms. Virus name acronyms are provided in Table 1.

N_3_ correction shuffled third positions of synonymous codons, thus precisely preserving genomic mononucleotide frequencies at the third codon position (Fig. 1). Technically, all synonymous third position nucleotides were first extracted from the genome to a pool, and then randomly distributed back among synonymous positions whilst preserving the amino acid sequence.

### (GC)_3_ correction

(GC)_3_ correction shuffled third positions of synonymous codons like N_3_ correction, but prior to distribution of the third-position nucleotides from the pool back to sequence G content was set equal to C, and A equal to T, therefore preserving combined GC content, but not individual nucleotide content.

### dN_23_ and dN_31_ corrections

dN_23_ and dN_31_ corrections similarly shuffled dinucleotides between positions 2-3 (or 3-1, respectively) of synonymous codons whilst preserving amino acid sequence; 6-fold degenerate codons were treated as independent 2- and 4-fold degenerate. As a result, first or second codon position was intact and only the third position was changed. Use of dN_23_ dinucleotide shuffling ensured precise preservation of position 2-3, but not position 3-1 dinucleotide content, while the dN_31_ had an opposite effect.

### dN_231_ correction

dN_231_ correction swapped compatible trinucleotides between codon positions 2-3-1 whilst preserving amino acid sequence (Fig. 1); 6-fold degenerate codons were treated as independent 2- and 4-fold degenerate. As a result, codon position 2 and position 1 of the following codon were identical before and after shuffling, and distinct genomic dinucleotide content at both codon positions 2-3 and 3-1 was the same as in the original sequence (Fig. 1).

### Relative dinucleotide bias

Relative dinucleotide bias (RDB) was calculated as the ratio of observed to expected dinucleotide frequency (the product of genomic nucleotide frequencies at the corresponding codon positions, e.g. £ [C_2_]×£ [G_3_] for CpG_23_ dinucleotide) to exclude the effect of mononucleotide composition on apparent dinucleotides ratio.

Statistical analysis was carried out with Student's t-test as implemented in Microsoft Excel. Spearman correlation was calculated with GraphPad Prism 4.0 (GraphPad Software Inc.). Variance of nucleotide frequencies was calculated as 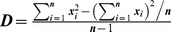, where ***n*** is the number of states (2 for GC content, 4 for mononucleotides, 16 for dinucleotides) and ***x*** is the frequency of a (di)nucleotide.

## Results

A well-known feature of genomes in general, and RNA viruses in particular, is dinucleotide bias [36], which also complements to CUB [34]. There are three dinucleotide positions in a codon. Variation of dinucleotide 1–2 is strongly restricted by amino acid sequence. Dinucleotides 2-3 and 3-1 may seem equally evolutionarily flexible because they both include the variable third codon position. Usually, a correction for either position 2-3 or 3-1 dinucleotide content is used to explore origins of CUB [37]. However, dinucleotide content varies between codon positions 2-3 and 3-1. To illustrate this, the frequency of the CpG dinucleotide at these codon positions was calculated in RNA viruses. As reported previously [36], CpG dinucleotide was suppressed in all RNA viruses, albeit to a different extent. Relative frequency of CpG dinucleotide was lower at codon positions 2-3 compared to 3-1 in 26 out of 29 RNA viruses (Fig. 1A). To evaluate the significance of this observation, extended datasets were tested for five viruses (Fig. 1B). In four viruses lower CpG frequency at codon position 2-3 was statistically significant with p-values below 10^−7^ (Student's t-test). In influenza A virus there was an insignificant excess of CpG at codon positions 2-3 (p>0.05), which could be explained, for example, by different selection pressures in the mammalian and bird hosts [38]. Statistically significant difference of RDB between codon positions could also be observed for other dinucleotides in many RNA viruses (data not shown), but without an obvious common pattern among the codon positions, as seen for the CpG dinucleotide.

We then evaluated how accounting for the complex dinucleotide composition patterns could improve analysis of codon usage bias. A general rationale for analyzing impact of (di)nucleotide bias on ENC is randomizing or shuffling synonymous codons in the original genome sequence whilst preserving the factor under study [37]. When shuffled sequences have an ENC close to the original sequence, the constraint (e.g. the nucleotide content) fully explains the CUB (or, in simple words, at that (di)nucleotide composition ENC cannot possibly be higher than it is). On the other hand, a difference between ENC in the original and simulated sequences shows the extent of CUB that could not be explained by the kind of pressure (nucleotide or dinucleotide) that was used as a constraint for the shuffling algorithm. Shuffling was preferred to randomization because it precisely preserved the positional dinucleotide content, produced sequences with more uniform ENC values, evident as lower standard deviation (SD) in sequences that were shuffled vs. randomized under the same constraint, and slightly lower ENC values than upon randomization (data not shown). Unfortunately, common statistical tests were poorly applicable to this approach, because all factors that affect codon preference (e.g. structural RNA elements) could not be accounted for, and therefore even a negligible difference in ENC between the original and generated sequences (e.g. 0.2 SD) passed a formal significance test upon increasing the number of replicates. As the standard deviation of ENC in scrambled sequences of a virus was on average 0.29 upon (GC)_3_ correction, 0.27 upon N_3_ correction,0.22 upon dN_23_ correction and 0.21 upon dN_231_ correction), and maximum SD in any virus/algorithm combination was 0.62, ENC difference above 1 was considered as significant.

Consistent with previous studies [34], ENC in RNA viruses ranged from 38.2 to 58.3. In sequences shuffled with preserved third codon position GC content, ENC was below 61 in all viruses (Fig. 1), indicating the effect of GC content on CUB. This finding was compatible with ENC values in RNA viruses that were observed upon Nc' correction, which is also based on GC content [34]. However, the ENC in (GC)_3_ shuffled sequences was higher than the original ENC on average by 3.5, but by up to 8.9, indicating that GC content imparity cannot explain all CUB observed in RNA viruses. Viruses often have uneven mononucleotide content, thus compromising GC correction approaches. For example, Aichi picornavirus has 54% C and only 16% G content at the third codon position [39].

Shuffling of the third-position nucleotide (N_3_ correction) produced ENC values lower than (GC)_3_ correction in all viruses. In some instances the difference between (GC)_3_ and N_3_ randomization was marginal, but in 8 out of 29 viruses it was above 1 and up to 4 in, e.g. HTLV1 and HEV. Overall, N_3_ shuffling, although an improvement over GC randomization, produced ENC values that were higher than the original ENC by more than one in all viruses except for Rubella, indicating residual unexplained bias.

**Figure 2:**
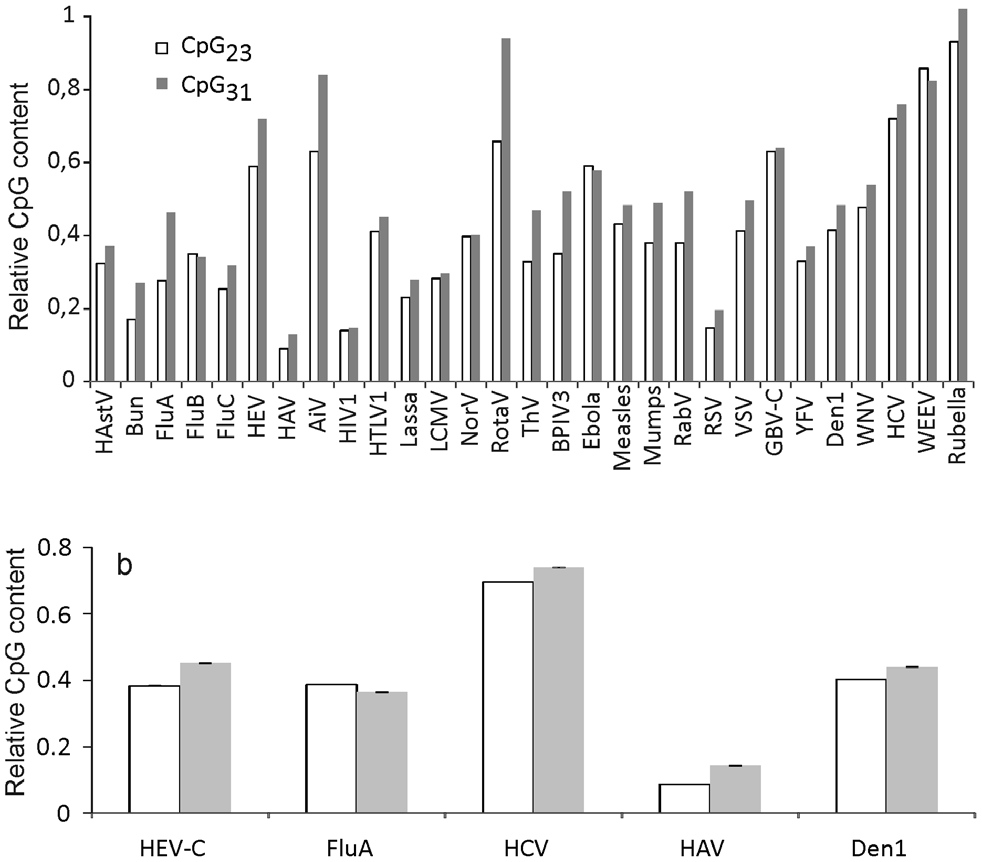
CpG content at different codon positions in RNA viruses. (A) Relative CpG dinucleotide content at codon positions 2-3 and 3-1 in RNA viruses (Table 1) was calculated as a ratio of the observed CpG content to the expected, calculated as the product of genomic nucleotide frequencies at the corresponding codon positions, e.g. £ [C_2_]×£ [G_3_] for CpG_23_ dinucleotide. (B) Mean relative CpG dinucleotide content in extended datasets of five RNA viruses. White, codon positions 2-3; gray, codon positions 3-1. Error bars indicate standard errors of the mean. Virus name acronyms are provided in Table 1.

The impact of dinucleotide content on CUB was next tested. A notable disparity of dinucleotide frequencies at codon positions 2-3 and 3-1 (Fig. 2) justified treating them independently in shuffling algorithms. In 22 out of 29 viruses, shuffling of codon position 2-3 dinucleotide (dN_23_) produced ENC values that were lower by more than 1 than ENC upon N_3_ correction (Fig. 1). In 15 viruses, dinucleotide content-corrected ENC values were lower than N_3_-corrected values by more than 2, indicating a strong impact of position 2-3 dinucleotide content on CUB. Shuffling of dinucleotide position 3-1, on the contrary, did not result in a decrease of ENC, which differed from N_3_ shuffled sequences by less than 0.3 SD in all viruses. To evaluate the impact of distinct dinucleotide content in codon positions 2-3 and 3-1 simultaneously, the codon position 2-3-1 triplet was shuffled between compatible codon pairs (dN_231_ correction, Fig. 1). Consistent with results of dN_23_ and dN_31_ shuffling, ENC upon dN_231_ shuffling did not differ significantly from dN_23_ shuffling, supporting negligible impact of dinucleotide content at codon positions 3-1 on synonymous codon preference.

**Figure 3:**
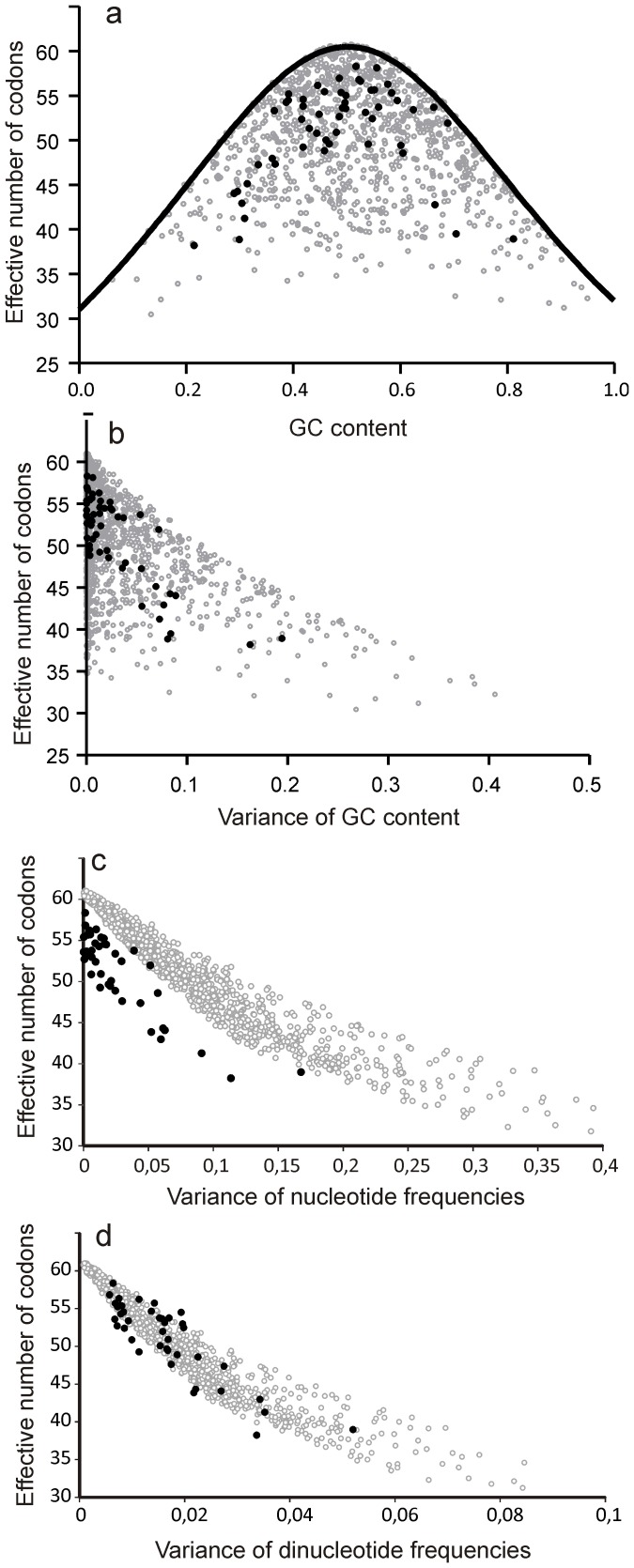
Plots of ENC vs. sequence content in 29 RNA viruses indicated in Table 1 (black dots) and in 1000 simulated sequences with random third-position nucleotide content (gray circles). Plot of ENC against GC content (A), variance of third-position GC content (B), variance of third-position nucleotide frequencies (D) and variance of dinucleotide frequencies at codon position 2-3 (D). Solid line in panel (a) indicates theoretical prediction of ENC as a function of GC content bias [31].

Shuffling synonymous codons is a practical way to approach origins of CUB. Another way to evaluate the effect of RNA composition on CUB is plotting ENC against GC content in studied sequences and comparing this to the theoretically expected ENC at various (GC)_3_ content [31]. In RNA viruses, actual ENC was always lower than the expected ENC at that GC content, implying that GC content does not fully explain CUB [34]. Unfortunately, the formula suggested by Wright to predict ENC values as a function of GC content [31] is not applicable to distinct frequencies of four nucleotides. To study limitations of classic GC content correction methods and develop a better measure of genome composition, a data set of one thousand 10002 nucleotide (3334 codon) long simulated coding sequences with highly variable third-position nucleotide content was generated. Firstly, relation of ENC and GC content in simulated sequences was compared to viral genomes and to the theoretical prediction of the Wright's curve [31]. While the theoretical estimation properly described maximum possible ENC as a function of GC content (Fig. 3A), it was not informative of a possible range of ENC at a given GC content in simulated sequences with extreme mononucleotide biases (Fig. 3A, grey dots). In fact, ENC values close to 30 could be achieved at any GC content. ENC in actual viruses was always below the Wright's curve (Fig. 3A, black dots); however the result for simulated sequences indicated that this is not necessarily an evidence of translational bias. Importantly, the limitations of GC content as the sole genome composition measure equally apply to eukaryotic genomes, as mRNAs may also have uneven mononucleotide composition [40]. Next, ENC in the simulated sequences was plotted against the variance of sequence content characteristics (Fig. 3B, 3C, 3D). Consistent with Figure 2A, GC content variance was a poor measure of sequence composition because it described only maximum ENC in simulated sequences, but poorly reflected possible ENC range at a given variance (Fig. 3B). Variance of mononucleotide and dinucleotide frequencies in simulated sequences provided a single measure of genome composition bias with a narrow corresponding ENC range (Fig. 3C, 3D, grey dots). Actual RNA virus sequences processed similarly (Fig. 3C, 3D, black dots) had notably lower ENC values than random sequences with the same variance of the third-position mononucleotide frequencies (Fig. 3C). This was consistent with results of mononucleotide content shuffling that only partially explained CUB (Fig. 1). When ENC in RNA viruses was plotted against the variance of dinucleotide frequencies at codon position 2-3 (Fig. 3D), the values overlaid with ENC values of simulated sequences, implying that dinucleotide bias can explain almost all apparent CUB in RNA viruses. Also, consistent with shuffling studies, in few viruses ENC was slightly lower than that of simulated sequences with the same variance of nucleotide content, indicating additional sources of CUB. Therefore, variance of dinucleotide frequencies is a good descriptor of mutational bias and can be used to predict a range of ENC for a given genome composition.

**Figure 4:**
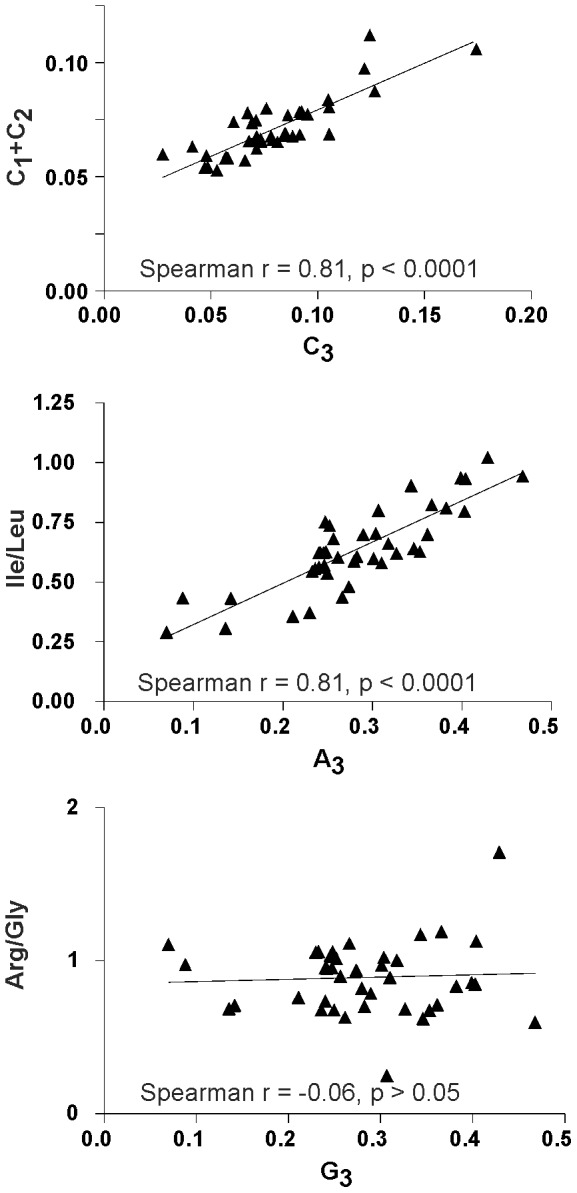
Correlation between sequence content at synonymous positions and sequence content at non synonymous positions/encoded protein content. Correlation between C content at codon positions 1+2 vs. 3 (A); ratio of isoleucine to leucine to third-position A content (B); ratio of arginine to glycine content to third-position G content (C) in 29 animal RNA viruses (Table 1).

This and previous studies identified mutational pressure, and the third-position nucleotide content in particular, as the main driver of synonymous codon preference. It has also been reported that GC content affects all codon positions in plant viruses [41] and can influence amino acid usage in HIV [42]. A plot of e.g. C content at codon positions 1+2 against position 3 in studied viruses (Fig. 4A) exemplifies that nucleotide content is generally consistent at different codon positions. A similar observation has been reported for vertebrate DNA viruses and plant viruses [41], [43]. A further analysis showed that genomic nucleotide content could significantly affect choice between compatible amino acids. The ratio of isoleucine (encoded by AUH triplets) to leucine (encoded by CUN and UUR triplets) in the viral protein sequence correlated with A content at the synonymous third codon position (Fig. 4B). Similar but weaker correlation could be observed between the third codon position G content and valine (GUN codons) to leucine (CUN and UUR codons) ratio (data not shown), but not for the ratio between the third-position G content and poorly compatible arginine (CGN and AGR codons) and glycine (GGN codons) content in the encoded protein (Fig. 4C). Previously, it has been shown that GC content in genomes of higher organisms can affect amino acid composition [44]–[46]. Our results indicate that this effect is maintained in RNA viruses. Therefore, genomic nucleotide content is not only the key driver of synonymous codon choice, but also a significant factor that defines choice between compatible amino acids.

## Discussion

The concept of synonymous and non-synonymous genetic variation is central to evolutionary studies. Synonymous, however, does not equal identical, because many factors can affect choice of synonymous codons. In RNA viruses “71% and 88% of CUB” was attributed to mutational pressure on nucleotide and dinucleotide content, respectively [34], which indicated the key role of mutational bias, but left a notable unexplained bias. A method that considered the impact of each of four nucleotides was superior to classical GC content correction (Figs. 1, 3), yet it failed to completely explain CUB in all but one virus. Dinucleotide bias at codon positions 2-3, but not 3-1, complemented notably to CUB in most viruses in our dataset. Overall, in 25 of 29 of the RNA viruses, mononucleotide and codon position 2-3 dinucleotide bias explained almost the entire apparent CUB. There is, however, a fundamental problem that complicates attributing dinucleotide bias to translational or mutational pressure. On one hand, in mammalian genomes there is CpG and UpA dinucleotide bias, and, consequently, NCG and NUA codons are relatively less frequent [47]. Therefore, translational bias towards common mammalian codons could produce secondary dinucleotide bias at codon positions 2-3. On the other hand, in RNA viruses there is also dinucleotide bias at codon positions 3-1 (Fig. 2), which implies additional types of pressure against, at least, CpG dinucleotide. Dinucleotide shuffling at distinct codon positions presented here allows distinguishing individual factors in a complex interplay of translational and mutational pressures on codon preference. Negligible effect of position 3-1 dinucleotide content on ENC (Fig. 1) may be explained by the fact that only few dinucleotides are usually highly biased, and e.g. pressure against the CpG dinucleotide at codon position 2-3 would strongly affect the four NCG codons and produce a strong bias. At positions 3-1 CpG bias would affect sixteen NNC codons, and only when they precede GNN codons. Therefore, the apparent effect of dinucleotide bias at codon positions 3-1 on CUB may be many times weaker that at codon positions 2-3, and below the detection limit of our method. Results of dinucleotide shuffling at codon positions 2-3 could also reflect the effect of translational bias, which could have produced secondary dinucleotide bias and increased dinucleotide content bias at codon position 2-3 compared 3-1 in most RNA viruses (Fig. 2). However, these are not the only possible explanations for the shuffling results.

Viruses are a unique form of life, and their life cycle that is largely distinct from host genome replication makes them both a valuable tool for exploring sources of CUB, and a source of confusion. In the mammalian host genome the CpG dinucleotide is underrepresented [48], because in this context C is methylated and then deaminated, producing C-T transition [14]. This creates an obvious mutational pressure on codon usage and results in underrepresentation of NCG codons. Such a feature of an organism's own genome allows recognition of non-self bacterial DNA by TLR9, an innate immunity sensor for CpG-rich DNA [18]. In mitochondria, CpG is also underrepresented [49], probably to avoid triggering TLR9. A similar sensor for CpG in RNA was predicted [38], but is not yet known. This hypothetical sensor or effector could explain CpG_31_ bias (Fig. 2) and also the dramatic effect of introduction of CpG dinucleotide at codon positions 3-1 on poliovirus viability [50]. If such a sensor was linked to translation, as, for example, translation-coupled endonucleases involved in no-go RNA decay [51], it could produce codon position-specific pressure on CpG dinucleotide content. Unfortunately, due to the host CUB driven by host mutational pressure, it is fundamentally impossible to unambiguously distinguish translational and mutational pressure on CUB in mammalian RNA viruses using only a computational approach.

Overall, dN_23_ and dN_231_ shuffling produced ENC values that differed from the original sequence by less than one in 25 of 29 viruses, indicating that we explored almost all types of pressure that affect overall codon usage. In three viruses (HIV, bunyavirus, rotavirus, and thogotovirus) ENC values upon any correction were still higher than ENC of the original sequence by more than 1. Therefore, additional factors are required to explain this residual CUB. In HIV, editing of cDNA by APOBEC3 could introduce additional genome composition bias [52]. One additional unaccounted factor could be the influence of (di)nucleotide bias on 6-fold degenerate codons. Unfortunately, it is not possible to prudently evaluate such an effect. Randomization of 6-fold degenerate codons would inevitably rely on inadequate estimates in genome composition, either nucleotide content at codon positions 1 and 2, which is dependent on amino acid sequence, or the third-position statistics, which would produce changes in positional (di)nucleotide content. Shuffling dinucleotides between different synonymous codon positions, on the other hand, would ruin the positional (di)nucleotide frequencies in the genome. The latter approach to 6-fold degenerate codons was not superior over the algorithms used here, moreover it could introduce additional bias evident as ENC values higher than upon dN_231_ correction or lower than in the original sequence (data not shown).

The algorithms presented here provide a substantial improvement in dissecting origins of codon usage bias in RNA viruses. It is obvious that corrections based on GC content are inadequate not only in viruses, but in any application, as conventional dsDNA genomes may have different genome composition biases for different genome strands [40]. As for dinucleotide composition, different codon positions should always be analyzed alongside, because they are affected by different kinds of pressure. Considering only dinucleotide content at positions 2-3 or 3-1, as well as combining different codon positions for analysis, can lead to false conclusions. Altogether, different shuffling algorithms allowed almost complete exploration of the correlation of nucleotide content and ENC in all but a few RNA viruses, but led to a conclusion that it is not possible to definitively discover forces affecting dinucleotide bias and CUB by only computational means. A number of additional questions remain unanswered in the field of synonymous variation in the genomes. Firstly, the origin of nucleotide bias that is strong enough not only to drive synonymous codon preference, but also to affect amino acid usage, remains largely unknown. Second, the source of primary dinucleotide content bias in RNA viruses remains obscure. Third, a single virus genome might not contain enough information to discover fine factors affecting CUB. Scanning window approaches working with an alignment of multiple genomes, akin to bootscanning, and the corresponding significance criteria, are required to analyze local CUB, while algorithms that properly address the synonymous variation in 6-fold degenerate codons and account for RNA secondary structure might further clarify the role of translation pressure. Such statistical methods, coupled with experimental analysis, can also elucidate fundamental steps of virus–cell interaction.

## References

[1] Bulmer M (1987) Coevolution of codon usage and transfer RNA abundance. Nature 325: 728–730. 2434856 10.1038/325728a0

[2] Pan A, Dutta C, Das J (1998) Codon usage in highly expressed genes of Haemophillus influenzae and Mycobacterium tuberculosis: translational selection versus mutational bias. Gene 215: 405–413. 9714839 10.1016/s0378-1119(98)00257-1

[3] Gutierrez G, Marquez L, Marin A (1996) Preference for guanosine at first codon position in highly expressed Escherichia coli genes. A relationship with translational efficiency. Nucleic Acids Res 24: 2525–2527. 8692691 10.1093/nar/24.13.2525 PMC145967

[4] Gouy M, Gautier C (1982) Codon usage in bacteria: correlation with gene expressivity. Nucleic Acids Res 10: 7055–7074. 6760125 10.1093/nar/10.22.7055 PMC326988

[5] Thanaraj TA, Argos P (1996) Ribosome-mediated translational pause and protein domain organization. Protein Sci 5: 1594–1612. 8844849 10.1002/pro.5560050814 PMC2143486

[6] Li GW, Oh E, Weissman JS (2012) The anti-Shine-Dalgarno sequence drives translational pausing and codon choice in bacteria. Nature 484: 538–541. 22456704 10.1038/nature10965 PMC3338875

[7] Tsai CJ, Sauna ZE, Kimchi-Sarfaty C, Ambudkar SV, Gottesman MM, et al (2008) Synonymous mutations and ribosome stalling can lead to altered folding pathways and distinct minima. J Mol Biol 383: 281–291. 18722384 10.1016/j.jmb.2008.08.012 PMC2628389

[8] Fernandez V, Zavala A, Musto H (2001) Evidence for translational selection in codon usage in Echinococcus spp. Parasitology 123: 203–209. 11510686 10.1017/s0031182001008150

[9] Chamary JV, Hurst LD (2004) Similar rates but different modes of sequence evolution in introns and at exonic silent sites in rodents: evidence for selectively driven codon usage. Mol Biol Evol 21: 1014–1023. 15014158 10.1093/molbev/msh087

[10] Hershberg R, Petrov DA (2008) Selection on codon bias. Annu Rev Genet 42: 287–299. 18983258 10.1146/annurev.genet.42.110807.091442

[11] Chamary JV, Parmley JL, Hurst LD (2006) Hearing silence: non-neutral evolution at synonymous sites in mammals. Nat Rev Genet 7: 98–108. 16418745 10.1038/nrg1770

[12] Chen SL, Lee W, Hottes AK, Shapiro L, McAdams HH (2004) Codon usage between genomes is constrained by genome-wide mutational processes. Proc Natl Acad Sci U S A 101: 3480–3485. 14990797 10.1073/pnas.0307827100 PMC373487

[13] Sharp PM, Stenico M, Peden JF, Lloyd AT (1993) Codon usage: mutational bias, translational selection, or both? Biochem Soc Trans 21: 835–841. 8132077 10.1042/bst0210835

[14] Lister R, Pelizzola M, Dowen RH, Hawkins RD, Hon G, et al (2009) Human DNA methylomes at base resolution show widespread epigenomic differences. Nature 462: 315–322. 19829295 10.1038/nature08514 PMC2857523

[15] Li M, Wang IX, Li Y, Bruzel A, Richards AL, et al (2011) Widespread RNA and DNA sequence differences in the human transcriptome. Science 333: 53–58. 21596952 10.1126/science.1207018 PMC3204392

[16] Kleinman CL, Adoue V, Majewski J (2012) RNA editing of protein sequences: a rare event in human transcriptomes. RNA 18: 1586–1596. 22832026 10.1261/rna.033233.112 PMC3425774

[17] Woo PC, Wong BH, Huang Y, Lau SK, Yuen KY (2007) Cytosine deamination and selection of CpG suppressed clones are the two major independent biological forces that shape codon usage bias in coronaviruses. Virology 369: 431–442. 17881030 10.1016/j.virol.2007.08.010 PMC7103290

[18] Krieg AM, Yi AK, Matson S, Waldschmidt TJ, Bishop GA, et al (1995) CpG motifs in bacterial DNA trigger direct B-cell activation. Nature 374: 546–549. 7700380 10.1038/374546a0

[19] Player MR, Torrence PF (1998) The 2–5A system: modulation of viral and cellular processes through acceleration of RNA degradation. Pharmacol Ther 78: 55–113. 9623881 10.1016/S0163-7258(97)00167-8 PMC7157933

[20] Washenberger CL, Han JQ, Kechris KJ, Jha BK, Silverman RH, et al (2007) Hepatitis C virus RNA: dinucleotide frequencies and cleavage by RNase L. Virus Res 130: 85–95. 17604869 10.1016/j.virusres.2007.05.020 PMC2186174

[21] Simmonds P, Tuplin A, Evans DJ (2004) Detection of genome-scale ordered RNA structure (GORS) in genomes of positive-stranded RNA viruses: Implications for virus evolution and host persistence. Rna 10: 1337–1351. 15273323 10.1261/rna.7640104 PMC1370621

[22] Arora R, Priano C, Jacobson AB, Mills DR (1996) cis-acting elements within an RNA coliphage genome: fold as you please, but fold you must!!. J Mol Biol 258: 433–446. 8642601 10.1006/jmbi.1996.0260

[23] Steil BP, Barton DJ (2009) Cis-active RNA elements (CREs) and picornavirus RNA replication. Virus Res 139: 240–252. 18773930 10.1016/j.virusres.2008.07.027 PMC2692539

[24] Weill L, James L, Ulryck N, Chamond N, Herbreteau CH, et al (2010) A new type of IRES within gag coding region recruits three initiation complexes on HIV-2 genomic RNA. Nucleic Acids Res 38: 1367–1381. 19969542 10.1093/nar/gkp1109 PMC2831325

[25] Sharp PM, Li WH (1987) The codon Adaptation Index–a measure of directional synonymous codon usage bias, and its potential applications. Nucleic Acids Res 15: 1281–1295. 3547335 10.1093/nar/15.3.1281 PMC340524

[26] Ikemura T (1985) Codon usage and tRNA content in unicellular and multicellular organisms. Mol Biol Evol 2: 13–34. 3916708 10.1093/oxfordjournals.molbev.a040335

[27] dos Reis M, Savva R, Wernisch L (2004) Solving the riddle of codon usage preferences: a test for translational selection. Nucleic Acids Res 32: 5036–5044. 15448185 10.1093/nar/gkh834 PMC521650

[28] Zhou J, Liu WJ, Peng SW, Sun XY, Frazer I (1999) Papillomavirus capsid protein expression level depends on the match between codon usage and tRNA availability. J Virol 73: 4972–4982. 10233959 10.1128/jvi.73.6.4972-4982.1999 PMC112541

[29] Hoeffler WK, Roeder RG (1985) Enhancement of RNA polymerase III transcription by the E1A gene product of adenovirus. Cell 41: 955–963. 4005953 10.1016/s0092-8674(85)80076-3

[30] Fradkin LG, Yoshinaga SK, Berk AJ, Dasgupta A (1987) Inhibition of host cell RNA polymerase III-mediated transcription by poliovirus: inactivation of specific transcription factors. Mol Cell Biol 7: 3880–3887. 2828918 10.1128/mcb.7.11.3880-3887.1987 PMC368055

[31] Wright F (1990) The ‘effective number of codons’ used in a gene. Gene 87: 23–29. 2110097 10.1016/0378-1119(90)90491-9

[32] Novembre JA (2002) Accounting for background nucleotide composition when measuring codon usage bias. Mol Biol Evol 19: 1390–1394. 12140252 10.1093/oxfordjournals.molbev.a004201

[33] Fuglsang A (2006) Accounting for background nucleotide composition when measuring codon usage bias: brilliant idea, difficult in practice. Mol Biol Evol 23: 1345–1347. 16679346 10.1093/molbev/msl009

[34] Jenkins GM, Holmes EC (2003) The extent of codon usage bias in human RNA viruses and its evolutionary origin. Virus Res 92: 1–7. 12606071 10.1016/s0168-1702(02)00309-x

[35] Marshak A (1950) Comparison of the base composition of nucleic acids of nuclei and cytoplasm of different mammalian tissues. Biol Bull 99: 332–333. 10.1086/BBLv99n2p321 14791474

[36] Karlin S, Doerfler W, Cardon LR (1994) Why is CpG suppressed in the genomes of virtually all small eukaryotic viruses but not in those of large eukaryotic viruses? J Virol 68: 2889–2897. 8151759 10.1128/jvi.68.5.2889-2897.1994 PMC236777

[37] Greenbaum BD, Levine AJ, Bhanot G, Rabadan R (2008) Patterns of evolution and host gene mimicry in influenza and other RNA viruses. PLoS Pathog 4: e1000079. 18535658 10.1371/journal.ppat.1000079 PMC2390760

[38] Greenbaum BD, Rabadan R, Levine AJ (2009) Patterns of oligonucleotide sequences in viral and host cell RNA identify mediators of the host innate immune system. PLoS One 4: e5969. 19536338 10.1371/journal.pone.0005969 PMC2694999

[39] Lukashev A, Drexler JF, Belalov I, Eschbach-Bludau M, Baumgarte S, et al (2012) Genetic variation and recombination in Aichi virus. J Gen Virol 10.1099/vir.0.040311-0 22377582

[40] Rispe C, Delmotte F, van Ham RC, Moya A (2004) Mutational and selective pressures on codon and amino acid usage in Buchnera, endosymbiotic bacteria of aphids. Genome Res 14: 44–53. 14672975 10.1101/gr.1358104 PMC314276

[41] Adams MJ, Antoniw JF (2004) Codon usage bias amongst plant viruses. Arch Virol 149: 113–135. 14689279 10.1007/s00705-003-0186-6

[42] Berkhout B, Grigoriev A, Bakker M, Lukashov VV (2002) Codon and amino acid usage in retroviral genomes is consistent with virus-specific nucleotide pressure. AIDS Res Hum Retroviruses 18: 133–141. 11839146 10.1089/08892220252779674

[43] Shackelton LA, Parrish CR, Holmes EC (2006) Evolutionary basis of codon usage and nucleotide composition bias in vertebrate DNA viruses. J Mol Evol 62: 551–563. 16557338 10.1007/s00239-005-0221-1

[44] Besemer J, Borodovsky M (1999) Heuristic approach to deriving models for gene finding. Nucleic Acids Res 27: 3911–3920. 10481031 10.1093/nar/27.19.3911 PMC148655

[45] Foster PG, Jermiin LS, Hickey DA (1997) Nucleotide composition bias affects amino acid content in proteins coded by animal mitochondria. J Mol Evol 44: 282–288. 9060394 10.1007/pl00006145

[46] Singer GA, Hickey DA (2000) Nucleotide bias causes a genomewide bias in the amino acid composition of proteins. Mol Biol Evol 17: 1581–1588. 11070046 10.1093/oxfordjournals.molbev.a026257

[47] Nakamura Y, Gojobori T, Ikemura T (2000) Codon usage tabulated from the international DNA sequence databases: status for the year 2000. Nucleic Acids Research 292. 10592250 10.1093/nar/28.1.292 PMC102460

[48] Lander ES, Linton LM, Birren B, Nusbaum C, Zody MC, et al (2001) Initial sequencing and analysis of the human genome. Nature 409: 860–921. 11237011 10.1038/35057062

[49] Shioiri C, Takahata N (2001) Skew of mononucleotide frequencies, relative abundance of dinucleotides, and DNA strand asymmetry. J Mol Evol 53: 364–376. 11675596 10.1007/s002390010226

[50] Burns CC, Campagnoli R, Shaw J, Vincent A, Jorba J, et al.. Introduction of CpG and UpA dinucleotides within and across synonymous capsid region codons differentially decreases replicative fitness.; 2010; St.Andrews, Scotland. pp. 68.

[51] Doma MK, Parker R (2006) Endonucleolytic cleavage of eukaryotic mRNAs with stalls in translation elongation. Nature 440: 561–564. 16554824 10.1038/nature04530 PMC1839849

[52] Liddament MT, Brown WL, Schumacher AJ, Harris RS (2004) APOBEC3F properties and hypermutation preferences indicate activity against HIV-1 in vivo. Curr Biol 14: 1385–1391. 15296757 10.1016/j.cub.2004.06.050

